# Conditions for minimal intelligence across eukaryota: a cognitive science perspective

**DOI:** 10.3389/fpsyg.2015.01329

**Published:** 2015-09-03

**Authors:** Paco Calvo, František Baluška

**Affiliations:** ^1^MINT Lab, Department of Philosophy, University of MurciaMurcia, Spain; ^2^IZMB, University of BonnBonn, Germany

**Keywords:** minimal intelligence, eukaryota, learning, memory, adaptive behavior, decision-making, problem solving

What is minimal intelligence? Generally speaking, our understanding of intelligence has to do with sets of biological functions of organisms that exhibit a degree of flexibility against contingencies in their environment-induced behavioral repertoire. In principle, sensory perception, sensory-motor coordination, basic forms of learning and memory, decision-making and problem solving, are all marks of minimal intelligence subject to scrutiny with the toolkit of the cognitive sciences. The bottom line is that an appraisal of the behavioral repertoire of eukaryotes, and of the organizational features that sustain it, resists an interpretation in reactive, non-cognitive, terms.

Despite the manifest diversity in the behavior of animals, plants, fungi and protists, and the functional specialization of different eukaryote cells, cellular organization based on the division into a nucleus and a cytoplasm allows for the genomic collaboration in the overall guidance of the response patterns to be observed, for example, in growth and development. However, understanding the expression of overt behavior at the level of its eukaryote cellular basis, or unearthing connections between behavior and genes, are but one piece of the puzzle. The research program requires, not only that we assess the cellular changes to be associated with, say, behavioral flexibility, but also the direct comparison across organisms with an eye to highlighting similarities and differences in the behavior of eukaryotes. The objective is ultimately to obtain a general picture of the capacity of organisms to solve problems in novel, often stressful, situations that enable them to deal with variable and complex environmental circumstances. By anchoring and comparing minimal, and yet robust, forms of behavior both functionally and structurally, the ability of organisms to learn from previous experiences, to predict future stresses, and to shape as well as to select suitable environments will be better appraised.

The fact that eukaryotes effectively exhibit minimal forms of intelligence might not be breaking news. In effect, the list of minimally intelligent organisms may well include *E.coli* and other prokaryotes (Lyon, 2007; Richardson, 2012, 2013). Possible examples of bacterial intelligence include communication, decision-making, cooperative behavior, and social intelligence as important survival strategies (Ben-Jacob et al., 2004; Hellingwerf, 2005; Lyon, 2007; Shapiro, 2007; Ben-Jacob, 2014). Coordination is needed, and cellular electrical excitability for the purpose of the transmission of information relies upon the capacity of organisms to conduct signals from receptor to effector sites. As a matter of fact, so-called neuroid conduction (“the propagation of electrical events in of non-nervous, nonmuscular cells,” see Mackie, 1970, p. 319) takes place in protists and plants. Animal nervous systems do organize signaling systems, ion channels, or synapses in more complex forms, but the basic components are already present in precursor organisms (see Calvo, forthcoming, and references therein).

Although minimal intelligence is likely to be present already in Eubacteria and Archaea (Crespi, 2001; Shapiro, 2007; Baluska and Mancuso, 2009), for present purposes we exclusively consider discussion of minimal intelligence within Eukarya. This includes both unicellular and multicellular organisms. These organisms have simple nervous systems or lack a nervous system altogether (Jennings, 1923). In order to maintain a sharp focus on minimal forms of intelligence, we exclude chordates (e.g., mammals, fish, reptiles, and birds).

How does research on minimal intelligence contribute to cognitive science? Our attempt is to:

- Parcel out a set of conditions for minimal intelligence across living systems;
- Understand what sort of behavioral and biological properties and capacities warrant the ascription of a form of minimal intelligence to eukaryotes; and
- Build-up the concept of minimal intelligence from simpler to more complex life forms.

Our strategy is orthogonal to other attempts found in the literature. Attempts inspired by “dual-process” theories (Evans and Frankish, 2009), for instance, assume that intelligence in general is implemented by two markedly different processing subsystems. One older subsystem (evolutionary speaking) puts us in close relation to our fellow non-human animals, and allows for basic tasks such as pattern-recognition. Another more recent subsystem would subserve abstract reasoning and other competencies of that ilk (Stanovich and West, 2000). Approaches of this sort mark a divide between minimal forms of intelligence, those that are implemented by a more primitive subsystem, and those of full blown intelligence that are implemented by a more recently evolved subsystem.

Our objective is to understand how much can be revealed about higher-level cognition *before* a dual-processing dichotomy needs to be called for in the first place. This does not imply that intelligence, writ large, may end up requiring a divide-and-rule strategy. That is an open question. We aim to assess how much of intelligent behavior can be accounted for by positing overarching sets of mechanisms which can be generally ascribed as cognition scales up. Our rationale is that, contrary to conventional wisdom, we do not understand “scaling up” itself as a problem (Calvo and Gomila, 2008). Rather we consider it as an opportunity to unearth underlying general principles; that in this way we can bring to light the shared building blocks that allow for the emergence of minimal and yet robust forms of intelligence across living systems.

By considering a diverse set of eukaryotes, stronger interferences can be drawn about minimal forms of sensory and perceptual capacities; simple forms of learning (associative and non-associative); varieties of memory; goal-oriented behavior; controlled movement; communication; decision-making; attention; problem solving; survival circuits linked to emotion; and related properties and capacities as realized by different organisms. This is true not only for *Drosophila, C. elegans, Aplysia, Arabidopsis*, and other models of choice with stars on the *Biology Walk of Fame* (Lihoreau et al., 2012), but also for organisms less studied and more simple (Moroz, 2009, 2014, 2015; Adamatzky, 2012, 2015; Reid et al., 2012; Kunita et al., 2014; Pagán, 2014). Also these organisms show minimal forms of intelligence based on shared physiology and behavioral traits across eukaryotic living systems (Jennings, 1923; LeDoux, 2012).

One nice example of this is the light-induced escape behavior shared between such diverse organisms as *Drosophila* larvae, nematode *C. elegans* as well as roots of *Arabidopsis* and maize (Ward et al., 2008; Xiang et al., 2010; Yokawa et al., 2011, 2013, 2014; Burbach et al., 2012; Bhatla and Horvitz, 2015). Feeding behavior of *C. elegans*, for instance, appears to be inhibited by hydrogen peroxide produced immediately after illumination (Bhatla and Horvitz, 2015). Similarly, roots of *Arabidopsis* produce hydrogen peroxide within few seconds after their exposure to light (Yokawa et al., 2011). Illumination stress induces effective light escape tropism in roots (Burbach et al., 2012; Yokawa et al., 2013, 2014). Similar light escape behavior is known to take place also in *Drosophila* larvae (Keene and Sprecher, 2012; Kane et al., 2013). It is intriguing that evolutionarily very distant organisms living underground in darkness use the same signaling molecule, reactive oxygen species, to change their behavior under illumination.

In the particular case of plants, we believe that unveiling why their behavior is so flexible may cast a new light on intelligence in general (Trewavas, 2005, 2009, 2014, 2015). Consider plant anticipatory behavior (Novoplansky, 2009; Shemesh et al., 2010). Our underlying working hypothesis is that adaptive plant behavior can only take place by way of a mechanism that predicts sensory states. The notion of anticipation, however, may come in a variety of forms (Calvo, submitted). Whereas according to weaker readings, anticipatory behavior may rely upon the capacity of the system to model internally environmental sources of stimulation, stronger forms of anticipation that explain away internal modeling cannot be discarded beforehand (Stepp and Turvey, 2010). We may consider “predictive coding” and “strong anticipation” as working hypotheses subject to empirical scrutiny. According to “predictive coding” (Friston, 2012), behavior is to be explained pro-actively. Under a predictive coding reading, a process of probabilistic inference allows animals to scan their surroundings (Kok et al., 2013), estimating the likelihood that some particular state of affairs is the source of stimulation. “Strong anticipation,” by contrast, maintains that predictive success does not involve modeling the future at any stage, but is rather a function of actual past behavior (Stepp and Turvey, 2010; Stepp et al., 2011). In the case of plants, understanding of anticipation in terms of predictive processing calls for studies of how plants *model* the environmental sources of stimulation. Behavior of plants may thus be equally interpreted pro-actively: plants may be able to estimate the likelihood that one particular state of affairs, and not another, is the cause of its sensory states. A comparative analysis with respect to other eukaryotic life forms is also equally welcome.

Plants' directional (tropisms) and non-directional (nastic) responses also probably provide a salient example. The number of growth and movement responses is highest in roots which show gravitropism, phototropism, thigmotropism, chemotropism, oxytropism, halotropism, electrotropism as well as stress avoidance and escape tropisms (Gilroy, 2008; Baluska et al., 2009; Baluška and Mancuso, 2013). Plants live in complex environments and their survival is dependent on reliably sampling critical parameters from their environment using their abundant plant-specific sensory systems, and with sensitivity to particular environmental contexts (Trewavas, 2005, 2009, 2014). In fact, plants and their roots sample more than 20 different parameters from their environment and integrate this complex sensory information online in order to mount appropriate behavioral responses (Knight et al., 1998; Baluska et al., 2009; Hodge, 2009; Trewavas, 2009; Karban and Shiojiri, 2010; Baluška and Mancuso, 2013; Karban et al., 2014; Karban, 2015). It is nonetheless not clear in what sense examples such as these illustrate minimal intelligence. To do so, we must discard the hypothesis that the reaction of plants, animals, fungi or protists to environmental inputs is fully accounted for in terms of hard-wired instincts.

Whether anticipation, as observed under tropistic, nastic or any other overt behavioral response in plants, is accounted for in model-based terms or not may have consequences for the way we understand anticipation in “higher” systems. Both empirical and theoretical research approaches on minimal intelligence are needed. It is our conviction that the study of simple forms of behavior from an integral cognitive science perspective, identifying the conditions for minimal intelligence across eukaryote, will allow us to have a more comprehensive picture of what cognition ultimately consists of.

## Conflict of interest statement

The authors declare that the research was conducted in the absence of any commercial or financial relationships that could be construed as a potential conflict of interest.
